# DCE-MRI and DWI Integration for Breast Lesions Assessment and Heterogeneity Quantification

**DOI:** 10.1155/2012/676808

**Published:** 2012-11-19

**Authors:** C. Andrés Méndez, Francesca Pizzorni Ferrarese, Paul Summers, Giuseppe Petralia, Gloria Menegaz

**Affiliations:** ^1^Dipartimento di Informatica, Universita degli Studi di Verona, Strada le Grazie 15, CA'Vignal, 37134 Verona, Italy; ^2^Divisione di Radiologia, Istituto Europeo di Oncologia, Via Ripamonti 435, 20141 Milano, Italy

## Abstract

In order to better predict and follow treatment responses in cancer patients, there is growing interest in noninvasively characterizing tumor heterogeneity based on MR images possessing different contrast and quantitative information. This requires mechanisms for integrating such data and reducing the data dimensionality to levels amenable to interpretation by human readers. Here we propose a two-step pipeline for integrating diffusion and perfusion MRI that we demonstrate in the quantification of breast lesion heterogeneity. First, the images acquired with the two modalities are aligned using an intermodal registration. Dissimilarity-based clustering is then performed exploiting the information coming from both modalities. To this end an ad hoc distance metric is developed and tested for tuning the weighting for the two modalities. The distributions of the diffusion parameter values in subregions identified by the algorithm are extracted and compared through nonparametric testing for posterior evaluation of the tissue heterogeneity. Results show that the joint exploitation of the information brought by DCE and DWI leads to consistent results accounting for both perfusion and microstructural information yielding a greater refinement of the segmentation than the separate processing of the two modalities, consistent with that drawn manually by a radiologist with access to the same data.

## 1. Introduction

Responses to cancer treatment are increasingly differentiated based not only on tumor type, but also on genetic and histochemical biomarkers. Exemplifying the progress in this respect is breast cancer. Biopsy-derived histological biomarkers offer high biological specificity and play an important role in determining the choice of chemotherapeutic agent. As different parts of a tumor often show different histological signatures or have evolved to different stages of tumor progression that may impact on their response to a given therapy, it is important to obtain a complete coverage of the tumor. Biopsies, however, are difficult to localize within the breast, are subject to sampling errors, and can seldom be repeated. Thus, there is growing clinical interest in the possible role of imaging to describe anatomical and physiological heterogeneity of tumors [1, 2].

Magnetic resonance imaging (MRI) methods such as dynamic contrast enhanced (DCE) and diffusion weighted (DW) MRI methods are amongst those of interest as they provide noninvasive digital biomarkers with good spatial coverage and repeatability [3]. DCE-MRI uses serial acquisition of images during and after the injection of intravenous contrast agent and has been shown to reflect tumor vascularity [4, 5]. DWI, on the other hand, generates images that are sensitized to water displacement at the diffusion scale and can be used to calculate a quantitative index reflecting the apparent freedom of diffusion (apparent diffusion coefficient (ADC)). Preclinical and clinical data show that ADC reflects regional cellularity [6–8].

DCE-MRI has a high sensitivity for breast cancer detection (89–100%), while DWI has shown utility in predicting suitable therapies and monitoring response [9]. A recognized weakness of DCE and DW-MRI is their lack of specificity between tumor types as overlap between the findings of benign and malignant lesions results in variable specificity (37–86%) [9]. This is not entirely surprising given that across cancer types the common features tend to include such processes as cell proliferation, angiogenesis, and necrosis. The ability of DCE- and DW-MRI to provide a spatial depiction of these anatomical and physiological conditions within a tumor makes them natural tools for probing tumor heterogeneity. The reporting of MRI has long relied on visual assessment of several scans having different contrasts, but in relation to breast cancer, few studies have exploited this inherently multiparametric data in a unified manner [10–12]. Moreover, the most recent works mainly address the problem of comparing and retrospectively integrating the contributions from the different modalities, without exploiting the conjunct information. Nevertheless, these works have highlighted the potential of combining DCE-MRI and DWI to differentiate the core of the tumor from peritumoral tissues and normal tissues and thus provide an indication of lesion heterogeneity [13].

In this work, we propose the multimodal integration of the information provided by DCE-MRI and DWI of breast cancer lesions for evaluating their heterogeneity, that is, to divide the lesion into zones that share certain similarity when using combined information coming from different imaging domains. The ultimate intention of this protocol is to allow a more extensive, reproducible characterization of heterogeneity in tumors that have been previously identified by a clinician.

In all previous reports on breast lesion segmentation the representation of DCE curves and ADC maps has been that of features in a vector space defined by the image values [14–17]. In this work a different approach is followed exploiting dissimilarity-based representations (DBR) [18]. The concept of dissimilarity-based representation consists of focusing on the contrast, or distance, between objects and of measuring it by a suitable criterion. The term *object* refers, in the present context, to the information represented by each particular voxel. This information need not be of a single type and in this case consists of both signal intensities (i.e., the time-intensity enhancement curve for DCE-MRI) and the ADC parameter value (derived from DW-MRI). A key concept in DBR is that of a *proximity relation* between two objects, which does not need to be explicitly represented in a feature space. Objects are characterized through pairwise dissimilarities; instead of using an absolute characterization of the objects by a set of features, problem-centric knowledge is used to define a measure that estimates the dissimilarity between objects. Here, both DCE and DWI contribute to such a measure leading to a novel multimodal approach to tissue characterization.

This paper is organized as follows.  Section 2 describes the pipeline including the clustering and registration processing steps.  Section 3 presents the results, which are then discussed in Section 4, and Section 5 derives conclusions.

## 2. Materials and Methods

**Figure 1:**
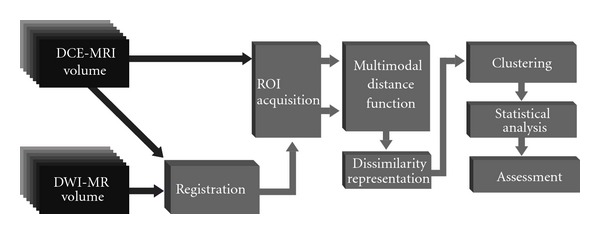
Perfusion/diffusion analysis and integration pipeline.

This section provides an overview of the pipeline shown in Figure 1 and details the methodological choices with respect to both clustering and registration. The DCE-MRI data are first visually inspected to identify a time point where the lesion has the higher contrast with respect to the surrounding tissue. Multimodal registration is carried out between DW-MRI and DCE-MRI images, allowing a spatial mapping of both volumes. Dissimilarity-based clustering is then performed integrating information from both acquisition modalities. Statistical analysis, consisting of nonparametric tests, were applied on the ADC distributions defined by the obtained clusters. An assessment of the results was carried out by clinical experts, and, for the sake of completeness, an evaluation of the tightness and separation of the clusters was also performed.

### 2.1. Multimodal Registration

 In order to perform voxelwise dissimilarity-based clustering that incorporates both DCE-MRI and DWI data, it is necessary to first spatially align the two datasets. The problem of registering between DCE-MRI and DWI becomes an increasingly difficult task in a highly compressible and elastic tissues like the breast, with its inhomogeneous anisotropic soft tissue, inherent nonrigid behavior, and lack of solid landmarks to guide the registration as fixed references. A standard registration protocol was used. Due to the highly distinct contrast and intensity characteristics of the two modalities as well as the low resolution of the DWI volumes, the registration process was divided into two steps, each following a standard multiresolution strategy. In the first step, rigid and affine transformations were performed successively in order to align and match the features of the fixed (DCE-MRI) and moving (DWI) images following a 5-level Gaussian scale space. In the second step a multiresolution cubic B-spline transformation with a regularization penalty was performed to elastically refine the alignment. Lesion-specific masks based on regions delineated by clinical experts were used in order to assign a greater weight to the voxels in the lesion area [19]. Normalized mutual information (NMI) was used as registration metric. In order to regularize the deformation, we used a bending energy penalty which is based on the spatial derivatives of the transformation [20]. The methodology used for registration was implemented in Elastix [19], and all the steps have been widely validated in literature [20, 21].

The registration protocol was applied to the b0 images from the DWI dataset and their transformation to the DCE-MRI space validated for each subject through visual inspection by an expert. The resulting transformation was applied to the remaining *b*-values, and the ADC was estimated on the transformed DWI images.

### 2.2. Dissimilarity-Based Clustering Methodology

The next step in the processing methodology is the construction of a dissimilarity matrix. This matrix consists of a set of row vectors, one for each voxel. These vectors represent the voxels in a vector space constructed by the dissimilarities to each other voxel. Usually, such a space can be safely treated as an Euclidean space equipped with the standard inner product definition.

Let *X* = {*x*_1_,…, *x*_*n*_} be a voxel-based dataset. Given a dissimilarity function, a data-dependent mapping *D* is defined as *D*(·, *R*) : *X* → *𝔻* ^*n*^ linking *X* to the so-called dissimilarity space [22]. The complete dissimilarity representation yields a square matrix consisting of the dissimilarities between all pairs of objects, such that every object is described by an *n*-dimensional dissimilarity vector *D*(*x*, *X*) = [*d*(*x*, *x*_1_) … *d*(*x*, *x*_*n*_)]^*T*^.

A distance function *D*_DCE_ based on the adaptive dissimilarity index first proposed in [23] has been exploited in a previous work [24] for calculating the pairwise proximity between DCE-MRI perfusion curves. There are two main approaches to quantifiably compare two time series: one makes use of the distances between the absolute values of their elements while the other focuses on the similarity of their behavior along time. Unlike conventional time-series distance functions, which focus only on the closeness of the values observed at corresponding points in time, ignoring the interdependence relationship between elements that characterize the time-series behavior, the proposed distance function takes into account the proximity with respect to values as well as the temporal correlation for the proximity with respect to behavior. For two voxel-derived perfusion curves *S*_1_ = (*u*_1_,…, *u*_*p*_) and *S*_2_ = (*v*_1_,…, *v*_*p*_), closeness with respect to behavior is defined as the combination of their monotonicity, that is, if both curves increase or decrease simultaneously, and the closeness of their growth rate over a determined period [23]. Both criteria are quantified by the temporal correlation present in the first term of the distance function *D*_DCE_, (1). The complete distance function *D*_DCE_ for DCE-MRI derived perfusion curves is defined as follows:

(1) $$\begin{array}{c} D_{\text{DCE}}\left(S_{1},S_{2}\right)=\frac{2}{1+\exp\left(\text{Cort}\left(S_{1},S_{2}\right)\right)}dH\left(S_{1},S_{2}\right), \end{array}$$

where *S*_1_ = (*u*_1_,…, *u*_*p*_) and *S*_2_ = (*v*_1_,…, *v*_*p*_) are two voxel-derived perfusion curves sampled at time instants (*t*_1_,…, *t*_*p*_) [23, 25]. Cort is the temporal correlation (2), and *dH* is the Hausdorff distance, defined in (3), which is used to measure the distance between both voxelwise perfusion curves:

(2) $$\begin{array}{c} \text{Cort}\left(S_{1},S_{2}\right)=\frac{\sum_{i=1}^{p-1}\left(u_{\left(i+1\right)}-u_{i}\right)\left(v_{\left(i+1\right)}-v_{i}\right)}{\sqrt{\sum_{i=1}^{p-1}{\left(u_{\left(i+1\right)}-u_{i}\right)}^{2}}\sqrt{\sum_{i=1}^{p-1}{\left(v_{\left(i+1\right)}-v_{i}\right)}^{2}}}, \end{array}$$

(3) $$\begin{array}{c} dH\left(S_{1},S_{2}\right)=\max\left\{\underset{u\in S_{1}}{\max}\;\underset{v\in S_{2}}{\min}||u-v||,\underset{v\in S_{2}}{\max}\;\underset{u\in S_{1}}{\min}||v-u||\right\}. \end{array}$$

The integration of the diffusion information into the dissimilarity function is accomplished through the addition of an ADC-dependent term *D*_ADC_ (4). This term is defined as a sigmoid function which makes use of the normalized difference between the ADCs (ADC_*S*1_ and ADC_*S*2_) of the voxels under consideration, which ranges from 0 to 1:

(4) DADC(S1,S2)=11+exp⁡(−kADC(||(ADCS1−ADCS2)/max⁡{ADCROI}||−0.5)).

**Figure 2:**
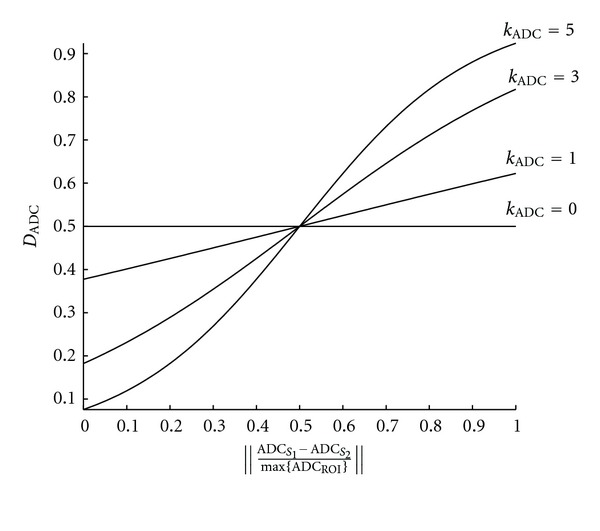
Effects of varying the tuning parameter *k*_ADC_ from (4).

The tuning parameter *k*_ADC_ weights the contribution of *D*_ADC_ to the complete dissimilarity measure *D* by modulating the shape of the sigmoid function. When the value of the normalized difference between ADCs is low, denoting similar ADC values between voxels, the dissimilarity function *D*_ADC_ approaches zero. On the contrary, when the value of the normalized difference between ADCs is high, denoting a large dissimilarity between ADC values between voxels, *D*_ADC_ approaches one, making the overall dissimilarity measure approaches the value of *D*_DCE_. The impact of the different values of *k*_ADC_ is illustrated in Figure 2.

The complete dissimilarity function *D* is then the product of *D*_ADC_ and *D*_DCE_:

(5) $$\begin{array}{c} D=D_{\text{ADC}}\cdot D_{\text{DCE}}. \end{array}$$

This global measure enables the monitoring of the performance as a function of the relative weight given to the ADC, as well as of different values of *k*_ADC_.

### 2.3. Performance Assessment

In each of the patients, a ROI was delineated by an expert around the lesion in the motion-corrected DCE-MRI volumes. Since unsupervised classification is sensitive to the general structure and distribution of the data, the ROI was drawn just exceeding the area of the enhancing lesion, allowing for a clear delineation of the heterogeneity of the lesion inside the ROI. The time-intensity curves normalized to the baseline at *t* = 0 and the corresponding ADC values from the voxels inside the ROI were treated as independent objects on a voxel by voxel basis. Using *D* from (5), a dissimilarity matrix was derived on a slicewise basis from the pairwise dissimilarities of the elements in the corresponding ROI. In such a space, each element was represented by a row vector whose dimensionality was defined by the cardinality of the ROI.

Once the dissimilarity space was constructed, the *K*-means algorithm [26] was used to group the voxels in the ROI into clusters. The initial centroids were calculated automatically following a preliminary clustering step with a random 10% subsample, as a strategy to improve the algorithm initialization avoiding a misplacement of the initial seeds. *K*-means minimize the sum over all clusters of the within-cluster sums of point-to-cluster-centroid distances using, in this case, the squared Euclidean distance.

For selecting the *K* number of clusters the standard clinical assessment protocol has been taken into consideration. It considers only three classes (persistent, plateau, and wash-out). An additional has been included for the surrounding tissue considering that the ROI exceeds the estimated limits of the enhancing lesion.

In order to perform a comparison with established methods the clustering procedure was also performed following a morphologic feature-based approach. This method relies on descriptors derived from the voxelwise time-intensity curves, comprising mainly specific characteristics of the shape of such curve. The features extracted from the DCE-MRI voxelwise time-intensity curves are baseline, maximum signal difference, time to peak, area under curve, maximum enhancement, wash-in rate, maximum slope of increase, wash-out rate, and the intercept of the line fitting the tail of the time-intensity curve with the axis *t* = 0. The use and definition of these morphologic features to describe the contrast agent intake can be found in the related literature [14, 17, 27]. Further, the clustering procedure was repeated incorporating the ADC of each voxel as an additional feature to the morphologic descriptor vectors calculated previously. The ADC and the morphologic features were standardized by subtracting their mean and dividing by their standard deviation. The results of these two procedures were compared with our method in order to assess the clustering and data representation outcome.

### 2.4. Patient Population

Data were acquired from 21 patients (age 50 ± 13.8 years). All the patients had been diagnosed to have primary ductal carcinoma.

DWI was acquired with a single-shot spin-echo (SE) echo planar imaging (EPI) sequence in three orthogonal diffusion encoding directions (*x*, *y*, and *z*) using 4 *b* values (0, 250, 500 and 1000 s/mm^2^) with parallel imaging (acceleration factor 2). Subjects were breathing freely, with no gating applied. The dataset consisted of 30 transverse slices (slice thickness 5 mm, no slice gap) and TR/TE 4800/71 ms, matrix 90 × 150 over the field of view (FOV) 184.5 × 307.5 mm.

DCE-MRI was performed using a 3D T1-weighted FLASH sequence (TR/TE 7.4/4.7 ms) with a flip angle of 25° and an acquisition matrix of 384 × 384 × 128 and field of view (FOV) 340 × 340 × 166 mm. Each 120-slice set was collected in 90 s at 8 time points for approximately 12 min of scanning. A catheter placed within an antecubital vein delivered 0.1 mmol/kg of the contrast agent, gadopentetate dimeglumine, (Magnevist, Wayne, NJ, USA) over 20 s (followed by saline flush) after the acquisition of one baseline dynamic scan. The DCE-MRI time series was motion corrected using the scanner manufacturer's in-line procedure.

## 3. Results

**Figure 3:**
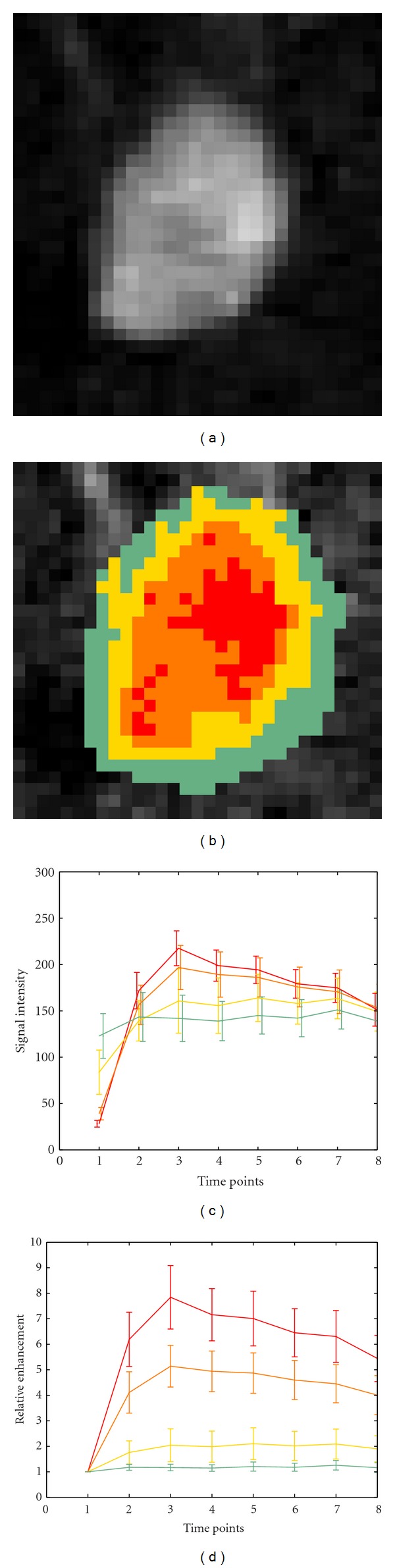
DCE-MRI image (a) and overlaid lesion clustering (b), comparison between the average raw (c), and normalized curves (d) calculated for each cluster.

 The regions resulting from dissimilarity-based clustering were rendered as colored overlays on the morphological images on each slice. The results from a representative patient are displayed in Figure 3. After clustering was performed on the normalized curves, the resulting clusters were assessed by the radiologists to validate the segmentation of both the central tumoral and surrounding regions.  Figure 3(b) shows examples of the clusters obtained, while Figures 3(c) and 3(d) represent the plots of the average time-intensity perfusion curves calculated on the raw and normalized data, respectively. The plots show the impact that the normalization step has in highlighting the intercluster differences. The central region exhibits a characteristic pattern in the DCE-MRI of a high early enhancement followed by a rapid washout, indicative of angiogenesis (Figure 3(d), red line). Typically, surrounding this central region lays a cluster featuring a pattern of rapid enhancement followed by a signal plateau (Figure 3(d), orange line). The outermost cluster surrounding these two central regions features a slow enhancement behavior (Figure 3(d), yellow line). The voxels corresponding to the each cluster were extracted from the spatially registered 3D ADC maps in order to perform statistical analysis. The analysis was carried out in the whole 3D ROI, that is, taking into account the ADC values corresponding to all the clustered slices as a single volume. Normality tests (Jarque-Bera) revealed that the ADC values for the different clusters analyzed were not normally distributed. Accordingly, a nonparametric test (Wilcoxon-signed-rank test) was used (*P* = 0.05) to evaluate whether the tumor's subregions corresponded to regions in the ADC maps with statistically different PDFs. In this way we found that the distributions of the ADC values in the DCE-MRI defined regions were statistically different, in each one of the two conditions, in 19 out of 21 patients.

The radiologist reviewed the overlays in comparison to the DCE seen as a dynamic loop, the DWI images, and the ADC maps derived from them, as well as T2 STIR images. Criteria for the review were whether or not any of the subregions obtained by the method corresponded to a zone of necrosis based on the complete set of images and whether one or more regions that would be classified as either benign or malignant have been subdivided.

**Figure 4:**
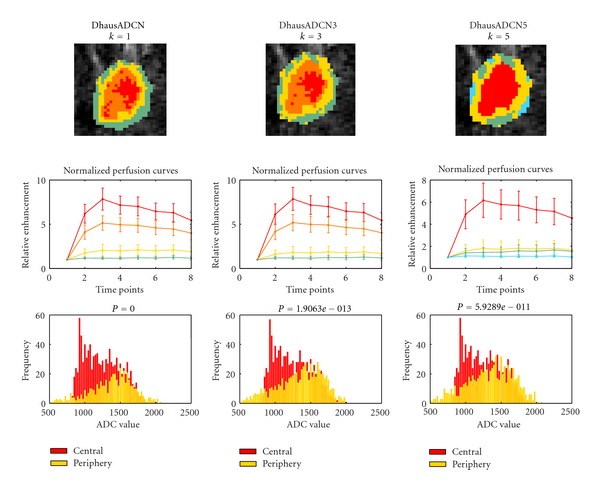
Clustering results using different values for the tuning parameter *k*_ADC_ (1, 3, and 5).

Figure 4 illustrates a typical case setting *k*_ADC_ to 1, 3, and 5. From the obtained results it was highlighted by the experts the usefulness of varying the parameter *k*_ADC_ to emphasize different characteristics of the lesion. A high *k*_ADC_ allows the discrimination between the core tumor and the surrounding regions by giving a higher weight to the difference between ADCs. This is mainly due to the fact that there is a progressive increase in ADC from the core of the tumor to peritumor tissues to normal tissues that leads to the possibility to use the ADC for locoregional staging [28]. Lowering *k*_ADC_ allows the subdivision of the core based on DCE-MRI dissimilarity and the evaluation of the heterogeneity of the tumor thanks to the balanced contribution of DCE and DWI in the distance function *D*.

For the sake of cluster comparison and validation among different methods, the silhouette analysis was used in all the clustering results. The silhouette analysis measures how close each point in one cluster is to points in the same cluster and how far away it is to points in the neighboring clusters. This is performed by quantitatively comparing the clusters by their tightness and separation and its average width provides an evaluation of cluster validity [29]. The silhouette analysis highlighted an improved performance of 31% for the clustering performed using *k*_ADC_ = 1 with respect to the established approach that employs morphologic features derived from the DCE-MRI time-intensity curves and the ADC as an additional feature (Table 1).

## 4. Discussion

 As a general strategy, we have demonstrated a dissimilarity clustering based on multidimensional data derived from diffusion and perfusion MRI. Extension of the algorithm to additional data is straightforward, though the computational demand rises, and the similarity metric will likely need to incorporate further context-specific knowledge. As examination of tumor heterogeneity is carried out on a tumor by tumor basis, the data space can be restricted to areas containing lesions already located, but not necessarily segmented. For the specific use of DCE and DW-MRI, the lower resolution of the DWI data presents an issue of partial volume effects that affects the clustering of small lesions, but this issue is not specific to any one characterization strategy.

The two free parameters of the protocol: number of clusters (*K*), and relative weighting of the diffusion data (*k*_ADC_), warrant discussion as the present work provides only a starting approximation to their choice, and the values may well be pathology dependent. For an unsupervised classification as used herein, the number of clusters should follow the actual structure and separation of the data into natural groups.

For breast tumors such as ductal carcinoma, the reporting of DCE-MRI data is currently based on a three-way division, while DWI is binary between normal and abnormal. The three DCE curve types (a rise and fall, a rise to a plateau, and a steady rise) have established clinical utility in predicting tumor malignancy [30]. This is not to say however that only three subgroups are possible, nor that these subgroupings are predictive of treatment response, which is the motive for examining tumor heterogeneity. In fact, works such as [14] have demonstrated that as the temporal resolution increases, a higher number of curve archetypes can be naturally identified and can be used for classification of voxelwise perfusion curves.

We consider it noteworthy, therefore, that when *K* was reduced to just three or four groups, these were identifiable with the 3 enhancement patterns (or these three and non-enhancement) used in clinical practice for the assessment of the breast cancer. As well, the confines of the groups with DCE-MRI time-course patterns consistent with malignant and benign tumors coincided very closely with the tumor margin drawn by a radiologist. Increasing the *K* value showed the expected progressive splitting of these groups as *K* increased, with *k*_ADC_ providing a distinction in the way this splitting proceeded based on the relative weight given to the diffusion data. The benefits of increasing the number of clusters are evident for understanding the heterogeneity of the lesion and the distribution of voxels that share certain similarities; however, the increase of the number of clusters should go hand to hand with cluster and data analysis techniques in order to avoid false or meaningless divisions. The overall protocol would also benefit from an integrating methodology such as cluster ensembles, in order to combine the multiple base clusterings done with different *k*_ADC_ values into a unified consolidated clustering, reaching with this a consensus solution.

The primary criteria for noninvasive assessment of tumors based on DCE MRI involve three enhancement patterns (four including necrosis/nonenhancement). In the clinical data used for this study this assessment criteria have limited the validation to the visual interpretation of enhancement patterns based on the conventional interpretation of DCE curves, with a reader-dependent incorporation of ADC information. Ultimately, the envisaged application is in anticipating and evaluating treatment response. If tumor heterogeneity in terms of both perfusion and diffusion is to be encompassed, the conventional 3-way categorization may not be adequate or appropriate and indeed for other organs this rating is less common. We are now looking into robust methods for further validation of the processing pipeline that would enable a clinical exploitation of the multimodal analysis. Access to ground truth beyond radiological and biopsy evaluation is needed and likely requires voxelwise comparison of with histology of resections, a process that requires modifications to the surgical procedure that were not justified for this first demonstration of the method. Even were histology image data available, a significant task remains in the spatially correlation of individual MRI voxels with the histological results in order to get the requisite voxel-scale validation.

## 5. Conclusions

 In this paper, we presented a general methodology for heterogeneity quantification that integrates information from diffusion (an indicator of cellularity) and perfusion (reflecting blood volume, flow, and vascular permeability) MRI images and illustrated its use in application to ductal carcinoma. The demonstration illustrated that multimodal clustering leads to improved selectivity and yields a greater refinement of the segmentation of tissues within the lesion than the separate processing of the two modalities.

By demonstrating that statistically consistent subgroups can be defined within tumors based on a combination of DCE-MRI and DWI-MRI data, we have indicated a means for objectively segmenting tumors that can be used for larger studies to examine clinical impact. Moreover, the appearance of statistically distinct perfusion regions within the tumor at moderate and low ADC weightings that in turn have statistically distinct ADC distributions suggests there is a useable distinction present that is not capitalized upon in present clinical practice.

## Figures and Tables

**Table 1:** Silhouette analysis scores describing cluster compactness and separation for the whole ROI and for each relevant region for the kinetic features and the multimodal lesion assessment (MMLA) methods (the higher the better).

Method	Mean	Central 1	Central 2	Periferic
Morphologic features	**0.51 **	0.53	0.51	0.49
Morphologic features + ADC	**0.47**	0.49	0.48	0.44
MMLA	**0.62**	0.57	0.65	0.61
MMLA + ADC, *k*_ADC_ = 1	**0.62**	0.57	0.64	0.62
MMLA + ADC, *k*_ADC_ = 3	**0.58**	0.54	0.59	0.60
MMLA + ADC, *k*_ADC_ = 5	**0.57**	0.56	0.58	0.58

## References

[1] Sharifi-Salamatian V, Pesquet-Popescu B, Simony-Lafontaine J, Rigaut JP (2004). Index for spatial heterogeneity in breast cancer. *Journal of Microscopy*.

[2] Yang X, Knopp MV (2011). Quantifying tumor vascular heterogeneity with dynamic contrast-enhanced magnetic resonance imaging: a review. *Journal of Biomedicine and Biotechnology*.

[3] Lloyd MC, Allam-Nandyala P, Purohit CN (2010). Using image analysis as a tool for assessment of prognostic and predictive biomarkers for breast cancer: how reliable is it?. *Journal of Pathology Informatics*.

[4] Knopp MV, Giesel FL, Marcos H, Von Tengg-Kobligk H, Choyke P (2001). Dynamic contrast-enhanced magnetic resonance imaging in oncology: theory, data acquisition, analysis, and examples. *Topics in Magnetic Resonance Imaging*.

[5] Su MY, Cheung YC, Fruehauf JP (2003). Correlation of dynamic contrast enhancement MRI parameters with microvessel density and VEGF for assessment of angiogenesis in breast cancer. *Journal of Magnetic Resonance Imaging*.

[6] Gibbs P, Liney GP, Pickles MD, Zelhof B, Rodrigues G, Turnbull LW (2009). Correlation of ADC and T2 measurements with cell density in prostate cancer at 3.0 Tesla. *Investigative Radiology*.

[7] Matsumoto Y, Kuroda M, Matsuya R (2009). In vitro experimental study of the relationship between the apparent diffusion coefficient and changes in cellularity and cell morphology. *Oncology Reports*.

[8] Jenkinson MD, Du Plessis DG, Smith TS, Brodbelt AR, Joyce KA, Walker C (2010). Cellularity and apparent diffusion coefficient in oligodendroglial tumours characterized by genotype. *Journal of Neuro-Oncology*.

[9] Partridge SC, DeMartini WB, Kurland BF, Eby PR, White SW, Lehman CD (2009). Quantitative diffusion-weighted imaging as an adjunct to conventional breast MRI for improved positive predictive value. *American Journal of Roentgenology*.

[10] Yankeelov TE, Lepage M, Chakravarthy A (2007). Integration of quantitative DCE-MRI and ADC mapping to monitor treatment response in human breast cancer: initial results. *Magnetic Resonance Imaging*.

[11] Jensen LR, Garzon B, Heldahl MG (2011). Diffusion-weighted and dynamic contrast-enhanced MRI in evaluation of early treatment effects during neoadjuvant chemotherapy in breast cancer patients. *Journal of Magnetic Resonance Imaging*.

[12] Yabuuchi H, Matsuo Y, Okafuji T (2008). Enhanced mass on contrast-enhanced breast MR imaging: lesion characterization using combination of dynamic contrast-enhanced and diffusion-weighted MR images. *Journal of Magnetic Resonance Imaging*.

[13] Yili Z, Xiaoyan H, Hongwen D (2009). The value of diffusion-weighted imaging in assessing the ADC changes of tissues adjacent to breast carcinoma. *BMC Cancer*.

[14] Lavini C, de Jonge MC, van de Sande MGH, Tak PP, Nederveen AJ, Maas M (2007). Pixel-by-pixel analysis of DCE MRI curve patterns and an illustration of its application to the imaging of the musculoskeletal system. *Magnetic Resonance Imaging*.

[15] Kuhl CK, Mielcareck P, Klaschik S (1999). Dynamic breast MR imaging: are signal intensity time course data useful for differential diagnosis of enhancing lesions?. *Radiology*.

[16] Degani H, Gusis V, Weinstein D, Fields S, Strano S (1997). Mapping pathophysiological features of breast tumors by MRI at high spatial resolution. *Nature Medicine*.

[17] Gal Y, Mehnert A, Bradley A, Kennedy D, Crozier S Feature and classifier selection for automatic classification of lesions in dynamic contrast-enhanced MRI of the breast.

[18] Pekalska E, Paclik P, Duin RPW (2002). A generalized kernel approach to dissimilarity-based classification. *Journal of Machine Learning Research*.

[19] Klein S, Staring M, Murphy K, Viergever MA, Pluim JPW (2010). Elastix: a toolbox for intensity-based medical image registration. *IEEE Transactions on Medical Imaging*.

[20] Rueckert D (1999). Nonrigid registration using free-form deformations: application to breast mr images. *IEEE Transactions on Medical Imaging*.

[21] Guo Y, Sivaramakrishna R, Lu CC, Suri JS, Laxminarayan S (2006). Breast image registration techniques: a survey. *Medical and Biological Engineering and Computing*.

[22] Pekalska E, Duin RPW (2005). *The Dissimilarity Representation for Pattern Recognition: Foundations and Applications*.

[23] Chouakria AD, Nagabhushan PN (2007). Adaptive dissimilarity index for measuring time series proximity. *Advances in Data Analysis and Classification*.

[24] Mendez CA, Pizzorni Ferrarese F, Summers P Multimodal MRI-based tissue classification in breast ductal carcinoma.

[25] Douzal-Chouakria A, Amblard C (2012). Classification trees for time series. *Pattern Recognition*.

[26] Xu R, Wunsch D (2005). Survey of clustering algorithms. *IEEE Transactions on Neural Networks*.

[27] Chen W, Giger ML, Bick U, Newstead GM (2006). Automatic identification and classification of characteristic kinetic curves of breast lesions on DCE-MRI. *Medical Physics*.

[28] Barcelo J, Vilanova JC, Luna A (2012). DWI of the breast. *Diffusion MRI Outside the Brain*.

[29] Rousseeuw PJ (1987). Silhouettes: a graphical aid to the interpretation and validation of cluster analysis. *Journal of Computational and Applied Mathematics*.

[30] American College of Radiology (2003). *Breast Imaging Reporting and Data System Atlas (BI-RADS Atlas)*.

